# Aberrant glycosylation of osteopontin in a rat renal stone formation model: A preliminary study

**DOI:** 10.1002/bco2.193

**Published:** 2022-09-26

**Authors:** Go Anan, Tohru Yoneyama, Takuo Hirose, Makoto Sato, Takefumi Mori, Chikara Ohyama

**Affiliations:** ^1^ Department of Urology Tohoku Medical and Pharmaceutical University Sendai Japan; ^2^ Department of Urology Yotsuya Medical Cube Tokyo Japan; ^3^ Department of Glycotechnology, Center for Advanced Medical Research Hirosaki University School of Medicine Hirosaki Japan; ^4^ Division of Nephrology and Endocrinology Tohoku Medical and Pharmaceutical University Sendai Japan; ^5^ Department of Urology Hirosaki University Graduate School of Medicine Hirosaki Japan

**Keywords:** aberrant, glycosylation, osteopontin, renal stone, calcium oxalate, inflammation

Nephrolithiasis is one of the most common diseases worldwide. Although the development of endoscopic surgery has expanded with minimally invasive surgery, effective medications for nephrolithiasis have not been established. Calcium oxalate (CaOx) is the most common component of nephrolithiasis. Thus, a better understanding of the molecular mechanisms underlying CaOx is required. Recent studies revealed that inflammation, including osteopontin (OPN), plays an important role in renal stone formation.^1^, ^2^ Previously, we first reported the aberrant glycosylation of OPN in the urine of patients with urinary stones; however, we could not examine their renal tissue.^3^ Therefore, the relationship between the aberrant glycosylation of OPN in renal tissue and renal stone formation is unknown. To the best of our knowledge, this is the first study to identify the glycosylation of OPN in renal tissue associated with renal stone formation. In this study, we used rats with ethylene glycol‐induced renal stones to evaluate the aberrant glycosylation of OPN in renal tissue.

An animal model of renal stone formation was generated using rats treated with ethylene glycol and alfacalcidol according to a previous report,^4^ with modifications (Figure 1). Sixteen 8‐week‐old male Sprague–Dawley rats were randomly divided into two groups, control rats (control) and renal stone formation rats (stone). The rats in the stone group were administered ethylene glycol (EG; 2.0 ml/kg body weight/day, subcutaneously; FUJIFILM Wako Pure Chemical, Osaka, Japan) and alfacalcidol (500 μg/kg body weight three times per week, administered orally; Chugai Pharmaceutical, Tokyo, Japan) to induce renal stones. As a control, the same volume of vehicle (saline instead of EG; 0.5% CMC instead of alfacalcidol) was administered. All rats were sacrificed on day 14, and their renal tissues (cortex and medulla) were collected.

**FIGURE 1:**
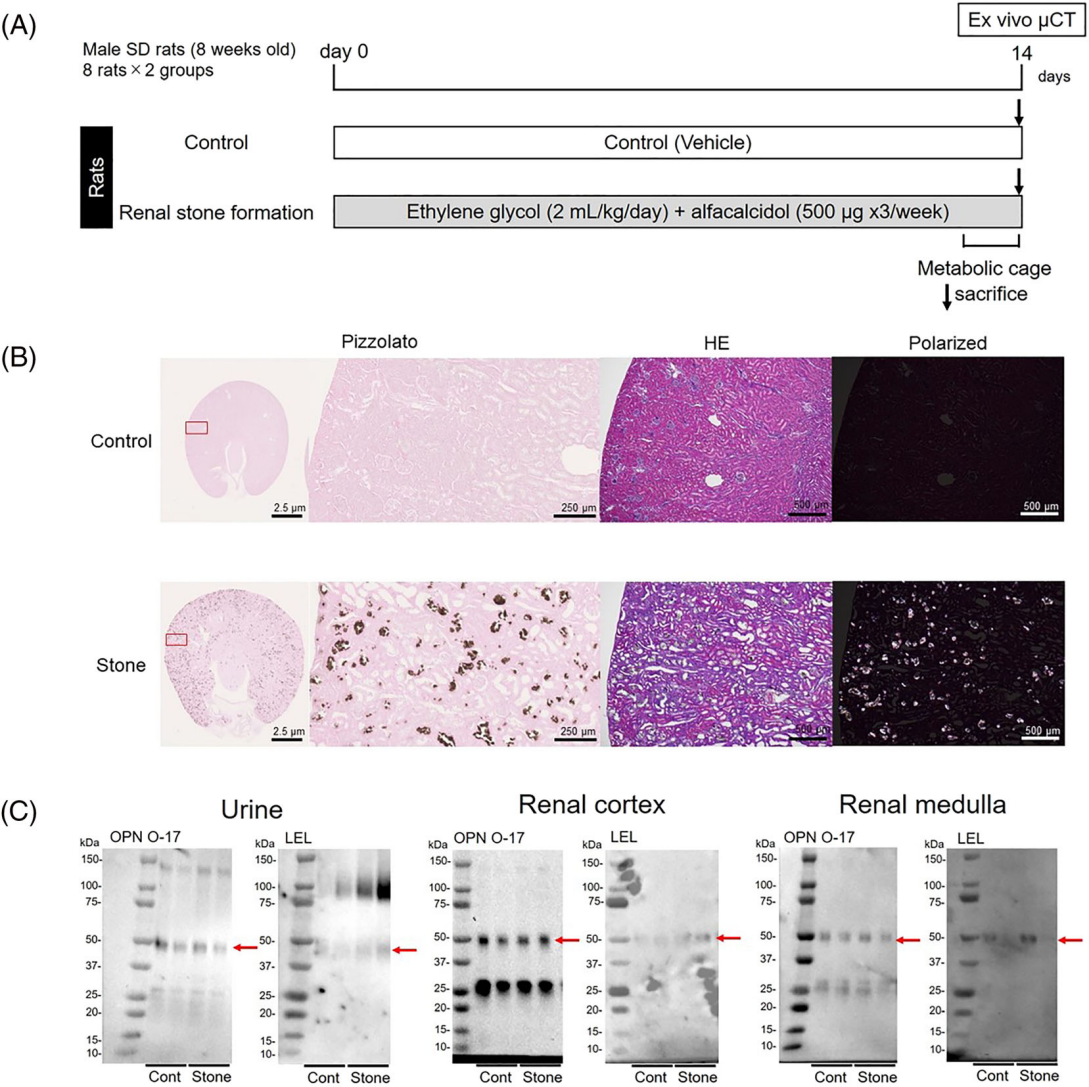
Rats with ethylene glycol‐induced renal stones to evaluate the aberrant glycosylation of osteopontin (OPN) in renal tissue. (A) Schematic diagram illustrating the induction of renal stone formation rats using ethylene glycol. (B) Representative normal and polarized photomicrographs of haematoxylin–eosin (HE) and Pizzolato staining in control and renal stone‐forming rats. (C) OPN and *Lycopersicon esculentum* lectin (LEL)‐reactive OPN in the urine, renal cortex, and renal medulla in control and renal stone‐forming rats

OPN immunoprecipitation, immunoblotting and *Lycopersicon esculentum* lectin (LEL) blotting were performed to search for glycosylated OPN, as described previously.^3^ LEL blotting revealed a poly‐*N*‐acetyllactosamine (polyLacNAc) glycan structure. Immunoblotting analysis was performed using an anti‐rat OPN antibody (O‐17; Immuno Biological Laboratories Co., Ltd., Gunma, Japan) and HRP‐conjugated secondary antibody. Lectin blotting analysis was performed using a Novex ECL Chemiluminescent Substrate Reagent Kit (Thermo Fisher Scientific Laboratories, Runcorn, United Kingdom).

Representative polarized photomicrographs of haematoxylin–eosin and Pizzolato staining in the control and stone groups are shown in Figure 1B. In the renal cortex, the amount of OPN was lower in the stone group than in the control group, whereas the level of LEL‐reactive OPN was higher in the stone group than in the control group (Figure 1C). In the renal medulla, there was no significant difference in LEL‐reactive OPN levels between the two groups. In the urine, the amount of LEL‐reactive OPN was increased in the stone group compared with that in the control group, whereas the OPN concentration was not different between the groups. The band at approximately 50 kDa represented full‐length OPN, whereas the band at 25–37 kDa was indicative of cleaved OPN. The difference in LEL at approximately 50 kDa indicated that full‐length OPN had LEL‐reactive glycosylation. In contrast, no reactive glycosylation of OPN was observed with cleaved OPN.

OPN plays a crucial role in renal stone formation.^1^, ^2^ Total knockout or mutation of the calcium‐binding site of OPN ameliorates glyoxylic acid‐induced CaOx crystal deposition in mouse kidneys.^5^ Therefore, OPN is an essential glycoprotein for urinary stone formation. LEL has been shown to react strongly with the polyLacNAc glycan structure.^6^ PolyLacNAc chain formation is an important function of endogenous lectin ligands, such as galectin.^7^ Determining changes in the OPN glycosylation profile of patients with renal stones is crucial as they might be related to OPN function. The decrease in OPN levels in the renal cortex of rats in the stone group, compared to that in the control group, might be because OPN was consumed during renal stone formation. Moreover, the LEL‐reactive glycosylation of full‐length OPN was observed in the stone group. The increase in the level of LEL‐reactive OPN, despite the decrease in total OPN levels, might be a marker of accelerated renal stone formation.

This study had several limitations. First, we attempted to use a lectin array; however, a sufficient amount of OPN could not be purified. Second, glycan structural analysis was not performed. Third, quantitative measurements of LEL‐reactive OPN were not possible owing to insufficient amounts of OPN; it is thus necessary to investigate the relationship between renal deposition and glycosylated OPN amounts in a further study. The present study showed that the levels of LEL‐reactive OPN were increased in the renal cortex and urine, even though total OPN levels did not increase. Our results are consistent with a previous report that showed the aberrant glycosylation of OPN in the urine of patients with urinary stones. These results suggest that the aberrant glycosylation of OPN occurs in the renal cortex and urine of rats with ethylene glycol‐induced renal stones and that LEL‐reactive OPN might be a marker of accelerated renal stone formation.

## AUTHOR CONTRIBUTIONS

Go Anan: conceptualization, data curation, investigation, and writing–original draft. Tohru Yoneyama: conceptualization, data curation, investigation, writing, review, and editing. Takuo Hirose: conceptualization, data curation, investigation, writing, review, and editing. Makoto Sato: supervision, writing, reviewing, and editing. Takefumi Mori: supervision, writing, reviewing, and editing. Chikara Ohyama: supervision, writing, review, and editing.

## CONFLICTS OF INTEREST

None declared.
